# Contrasting Leaf Adaptation Strategies of *Pinus koraiensis* and *Fraxinus mandshurica* Under Water and Nutrient Variation

**DOI:** 10.3390/plants15132053

**Published:** 2026-07-02

**Authors:** Jianfei Yang, Kangjing Lu, Haibo Wu, Mengguang Han, Hailong Shen

**Affiliations:** 1 College of Forestry, Northeast Forestry University, Harbin 150040, China; yjf19910304@126.com (J.Y.); 17613835661@163.com (K.L.); 2 Korean Pine Key Laboratory of the State Forestry and Grassland Administration, Northeast Forestry University, Harbin 150040, China; whb152407@163.com; 3 College of Ecology, Northeast Forestry University, Harbin 150040, China

**Keywords:** *Pinus koraiensis*, *Fraxinus mandshurica*, water regimes, anatomical traits, photosynthetic response

## Abstract

To clarify interspecific differences in the adaptive mechanisms of *Pinus koraiensis* and *Fraxinus mandshurica* seedlings to water–nutrient conditions, we compared their leaf morphological, anatomical, and photosynthetic responses under contrasting water and fertilization regimes. A pot experiment was conducted using three water levels: drought (DR, 30% of field capacity), control (CK, 55% of field capacity), and water addition (W, 85% of field capacity), combined with two fertilization treatments: no fertilization (NF) and nitrogen–phosphorus fertilization (F). Leaf morphology, anatomy, and photosynthetic traits were systematically measured and analyzed. The results showed that water availability was the dominant factor regulating seedling responses in both species, including increasing water supply generally promoting leaf expansion, photosynthetic rate, and anatomical development, whereas fertilization mainly exerted a water-dependent modifying effect. Under improved water supply, *P. koraiensis* exhibited a more conservative strategy, characterized by greater investment in conductive tissues and a threshold response of water-use efficiency (WUE). In contrast, *F. mandshurica* showed a more acquisitive strategy, with coordinated increases in leaf expansion, photosynthetic performance, and assimilatory–vascular tissue development under improved water conditions. These results indicate that both species share a common water-driven response pattern. However, they differ markedly in the coordination of morphological, physiological, and anatomical adjustments, reflecting contrasting resource-use strategies that should be considered in species-specific nursery water and fertilizer management.

## 1. Introduction

Climate change is reshaping water and nutrient regimes in terrestrial ecosystems [1]. The interactive effects of water availability and nutrient deposition on plant growth and physiological processes are critical for understanding forest ecosystem dynamics, particularly in regions sensitive to climate change. In temperate forests, drought and nutrient stress, especially nitrogen (N) and phosphorus (P) deposition, pose significant challenges to seedling regeneration and forest ecosystem stability [2,3,4]. In Northeast China, the broad-leaved Korean pine mixed forest (BKF) is increasingly exposed to the combined effects of water fluctuation and nutrient enrichment. As the dominant tree species and a key associated species in this ecosystem, *Pinus koraiensis* and *Fraxinus mandshurica* play key roles in stand regeneration, successional dynamics, and ecosystem functioning [5,6,7]. Therefore, understanding how their seedlings respond to coupled variation in water and nutrient availability is essential for clarifying the regeneration ecology of BKF and for improving nursery management and forest restoration practices.

Leaf morphological traits such as leaf area (LA), leaf width (LW), and specific leaf area (SLA) reflect both light interception potential and the trade-off between resource acquisition and conservative resource use [8]. Previous studies have shown that drought generally reduces leaf size and SLA while increasing tissue compactness or leaf thickness, thereby limiting water loss and enhancing water-use efficiency (WUE) [9,10]. In contrast, adequate water supply usually promotes larger leaves and higher SLA, indicating a more acquisitive growth strategy. Fertilization can further influence leaf morphology by regulating cell division, tissue expansion, and carbon–nutrient allocation [11,12]. However, in many cases, its effects are strongly dependent on water availability: under sufficient water, fertilization often promotes leaf expansion more clearly, whereas under drought, it may only partly alleviate growth inhibition by improving resource allocation [13].

Photosynthetic physiology constitutes the key functional link between environmental variation and seedling growth performance. Water deficit generally leads to significant declines in net photosynthetic rate (Pn), transpiration rate (Tr), and stomatal conductance (Gs), although the magnitude and mechanisms of these responses differ among species and provenances. In some cases, drought-induced reductions in photosynthesis are mainly driven by stomatal closure and the resulting limitation of CO_2_ diffusion [14,15]; in others, severe drought additionally causes non-stomatal limitation through damage to chloroplast structure, reduced photosynthetic enzyme activity, and impaired biochemical metabolism [16,17,18]. By contrast, appropriate fertilization often enhances photosynthesis by improving nutritional status, chlorophyll accumulation, and photosynthetic enzyme activity, thereby increasing carbon assimilation and resource-use efficiency [19,20]. However, the positive effects of fertilization on photosynthetic traits do not occur independently, but are strongly modulated by water conditions.

Leaf anatomical structure provides the structural basis for plant adaptation to variation in water and nutrient environments and represents the microscopic expression of morphological and physiological adjustment [21]. Under drought, plants often reduce water loss by increasing leaf thickness, palisade development, the palisade-to-spongy tissue ratio, and vascular development, whereas under an adequate water supply, they tend to form thinner leaves with more developed spongy tissues to enhance gas exchange and light-use efficiency [22]. Fertilization can also significantly affect leaf anatomy by regulating cell division, differentiation, and vascular development, thereby improving water and nutrient transport efficiency [23,24].

Under ongoing climate change, fluctuations in water availability and increasing nitrogen and phosphorus deposition are likely to jointly influence seedling establishment, resource allocation, and species regeneration in this forest ecosystem. However, previous studies have paid insufficient attention to integrated responses under a continuous water gradient in combination with N and P inputs, particularly for direct comparisons between co-occurring tree species with contrasting functional types and ecological strategies. This limitation is particularly evident in studies of BKF regeneration, where the seedling responses of *P. koraiensis* and *F. mandshurica* to coupled water–nutrient variation remain poorly understood. We hypothesized that *P. koraiensis* and *F. mandshurica* would differ in the coordination of leaf adaptation strategies under coupled water and nutrient conditions because of their contrasting functional strategies. Therefore, the present study systematically compared the responses of the two species in terms of leaf morphology, photosynthetic physiology, and leaf anatomical structure to different water regimes and nitrogen–phosphorus addition. By clarifying the coordinated mechanisms underlying seedling adaptive strategies, this study is expected to improve our understanding of seedling regeneration processes and to provide a theoretical basis for forest cultivation, precision water–fertilizer management, and the ecological restoration of degraded forests in Northeast China.

## 2. Results

### 2.1. Effects of Water Regime and Fertilization on Leaf Morphological Traits of Pinus koraiensis and Fraxinus mandshurica Seedlings

Water regime significantly affected leaf area (LA), specific leaf area (SLA), and leaf width (LW) in *P. koraiensis* seedlings (*p* < 0.05), whereas fertilization and the water × fertilization interaction significantly affected only LA (Table 1). The leaf expansion of *P. koraiensis* increased with increasing water supply, and LW showed a similar pattern. Under sufficient water conditions, fertilization significantly increased both LW and LA. In contrast, SLA decreased with increasing water supply under NF and F, suggesting that *P. koraiensis* tended to form relatively denser needles under higher water availability.

Water regime also significantly affected leaf morphology in *F. mandshurica* seedlings (*p* < 0.05) (Table 2). Similar to *P. koraiensis* seedlings, the LA and LW in *F. mandshurica* seedlings increased with increasing water supply. However, unlike *P. koraiensis*, SLA in *F. mandshurica* showed an increasing trend with water supply, although the differences were not significant. This indicates that the response of *F. mandshurica* to increased water supply was expressed mainly through overall leaf expansion rather than through substantial changes in leaf area per unit biomass. Furthermore, neither fertilization nor the water × fertilization interaction had significant effects on LA, SLA, or LW in *F. mandshurica* (*p* > 0.05), suggesting that variation in leaf morphology in this species was driven primarily by water availability.

### 2.2. Effects of Water Regime and Fertilization on Photosynthetic Parameters of Pinus koraiensis and Fraxinus mandshurica Seedlings

**Figure 1:**
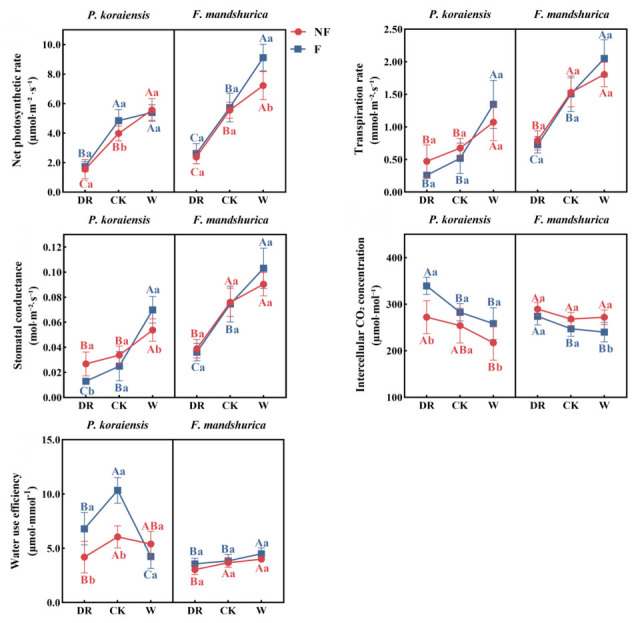
Effects of fertilization and water availability on photosynthetic performance and WUE in *Pinus koraiensis* and *Fraxinus mandshurica* seedlings. Different lowercase letters indicate that there are significant differences between the fertilization treatments under same water condition (*p* < 0.05), while the uppercase letters indicate that there are significant differences between the water treatments under fertilization or no fertilization condition (*p* < 0.05). Data are presented as mean ± SE (*n* = 5).

Water regime had highly significant effects on all photosynthetic parameters in both *P. koraiensis* and *F. mandshurica* seedlings (*p* < 0.01), confirming that water availability was the dominant factor controlling photosynthetic performance (Figure 1). In *P. koraiensis*, Pn, Tr, and Gs all increased markedly with increasing water supply, while Ci declined. Under F conditions, Pn, Ci, and WUE under CK and DR were significantly higher than those under NF conditions, whereas Tr and Gs were lower. These results indicate that fertilization can, to some extent, enhance WUE by reducing Tr while increasing Pn, thereby alleviating the negative effects of insufficient water on leaf growth.

A similar pattern was observed in *F. mandshurica*, where Pn, Tr, and Gs increased, and Ci decreased with increasing water supply. Unlike *P. koraiensis*, WUE in *F. mandshurica* increased continuously with water supply. Under W condition, fertilization further increased Pn by 26.31% and reduced Ci by 11.75%, indicating that nutrient addition mainly enhanced photosynthetic efficiency under well-watered conditions.

### 2.3. Effects of Water Regime and Fertilization on Anatomical Traits of Pinus koraiensis and Fraxinus mandshurica Seedlings

**Figure 2:**
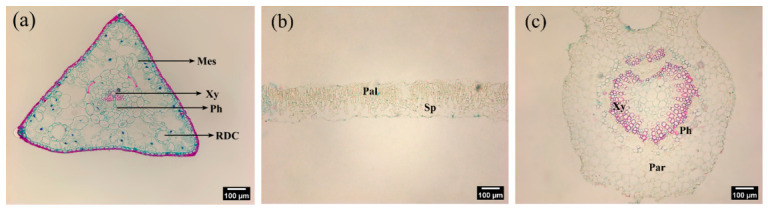
Anatomy of current-year needles of *Pinus koraiensis* (**a**) and current-year leaves of *Fraxinus mandshurica* (**b**,**c**). Note: RDC, resin duct cavity; Mes, mesophyll; Xy, xylem; Ph, phloem; Pal, palisade tissue; Sp, spongy tissue; Par, parenchyma.

Anatomical observations of leaves showed clear structural differences between *P. koraiensis* and *F. mandshurica* (Figure 2). In *P. koraiensis*, needle cross-sections were mainly composed of the epidermis, resin ducts, mesophyll tissue, xylem, and phloem. By contrast, *F. mandshurica* exhibited the typical anatomy of a broadleaf species, with well-differentiated veins composed mainly of parenchymatous and vascular tissues, and mesophyll clearly differentiated into palisade and spongy tissues.

**Figure 3:**
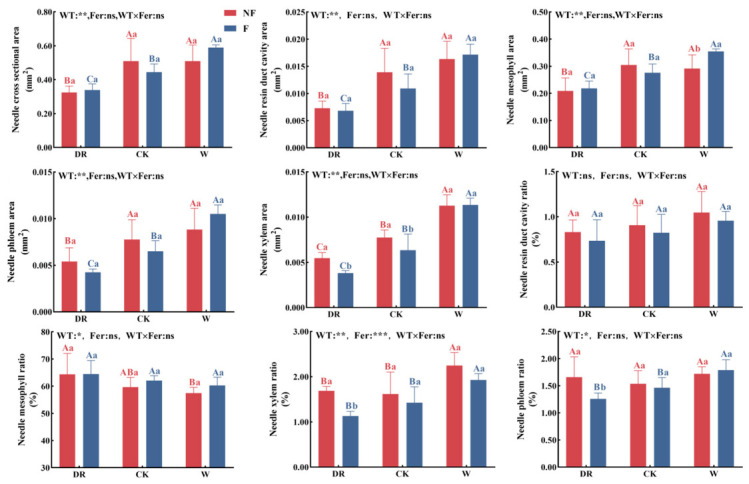
Leaf anatomical traits of *Pinus koraiensis* seedlings under different water and nitrogen–phosphorus fertilization treatments. Different lowercase letters indicate that there are significant differences between the fertilization treatments under same water condition (*p* < 0.05), while the uppercase letters indicate that there are significant differences between the water treatments under fertilization or no fertilization condition (*p* < 0.05). Data are presented as mean ± SE (*n* = 5). Asterisk represents water and fertilization has a significant effect on anatomical characteristics of needles. ns represents no significant. * indicates *p* < 0.05; ** represents *p* < 0.01, *** represents *p* < 0.001. WT, water treatment; Fer, fertilization treatment.

Water availability was the dominant factor shaping leaf anatomy in both species. In *P. koraiensis*, water treatment significantly affected all needle anatomical traits (*p* < 0.01), while fertilization significantly affected only the xylem ratio (Figure 3 and Figure S1). Under both F and NF conditions, water addition markedly increased the needle cross-sectional area, resin duct lumen area, mesophyll area, phloem area, and especially xylem area. At the same time, the xylem ratio increased, whereas the mesophyll ratio decreased under W, indicating that improved water supply not only enlarged the needles but also shifted structural investment toward conductive tissues. Fertilization effects were limited, although mesophyll area increased by 21.70% under W, while under DR, xylem ratio and phloem ratio declined by 32.91% and 24.06%, respectively.

**Figure 4:**
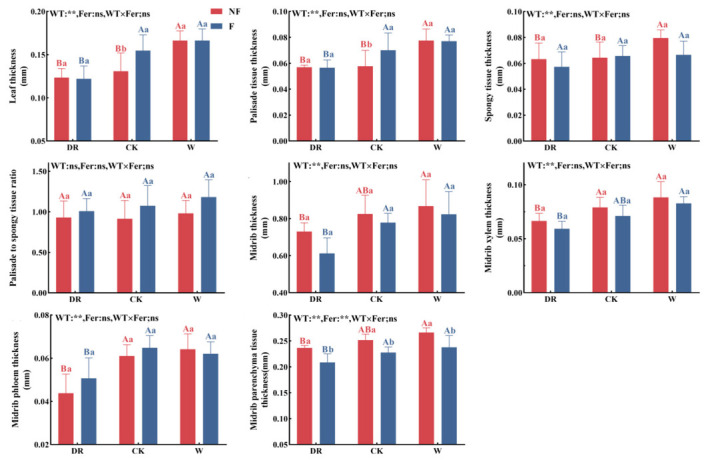
Leaf anatomical traits of *Fraxinus mandshurica* seedlings under different water and nitrogen–phosphorus fertilization treatments. Different lowercase letters indicate that there are significant differences between the fertilization treatments under same water condition (*p* < 0.05), while the uppercase letters indicate that there are significant differences between the water treatments under fertilization or no fertilization condition (*p* < 0.05). Data are presented as mean ± SE (*n* = 5). Asterisk represents water and fertilization has a significant effect on anatomical characteristics of needles. ns represents no significant. ** represents *p* < 0.01. WT, water treatment; Fer, fertilization treatment.

A similar water-driven pattern was observed in *F. mandshurica*, but its anatomical response was expressed mainly through leaf thickening and vein development (Figures S2 and S3). With increasing water supply, leaf thickness, palisade tissue thickness, spongy tissue thickness, and main vein tissues all increased (Figure 4). These results indicate that higher water availability promoted both assimilatory tissue development and vascular support in *F. mandshurica*. Fertilization effects were again weak: under CK, leaf thickness and palisade thickness increased by 18.28% and 21.31%, respectively, but main vein parenchyma thickness decreased by 11.87–9.62% compared with the NF treatment.

Overall, *P. koraiensis* responded to increased water mainly by enhancing conductive tissue investment within the needle, whereas *F. mandshurica* responded primarily through leaf thickening and stronger vein development. This suggests that *P. koraiensis* tended to reinforce internal transport capacity, while *F. mandshurica* showed a more coordinated expansion of assimilatory and supportive tissues under improved water conditions.

### 2.4. Correlations Among Leaf Morphological, Photosynthetic, and Anatomical Traits in Pinus koraiensis and Fraxinus mandshurica

**Figure 5:**
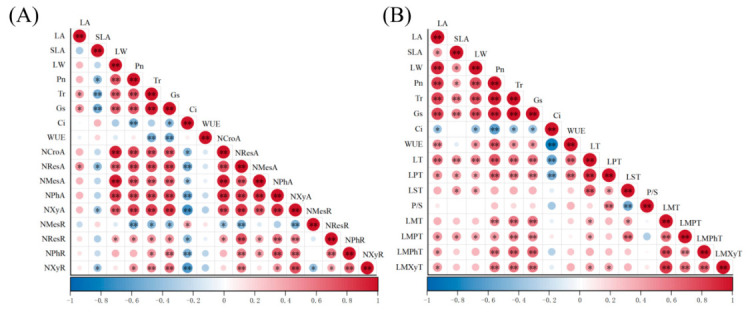
Correlation analysis of leaf traits in *Pinus koraiensis* (**A**) and *Fraxinus mandshurica* (**B**). NCroA, NMesA, NResA, NPhA, and NXyA represent the needle cross-sectional area, mesophyll area, resin duct lumen area, phloem area, and xylem area of *P. koraiensis*, respectively. NMesR, NResR, NStR, NPhR, and NXyR represent the mesophyll ratio, resin duct lumen ratio, stele ratio, phloem ratio, and xylem ratio of *P. koraiensis* needles, respectively. Red indicates a positive correlation, whereas blue indicates a negative correlation. LT, LPT, LST, and P/S represent leaf thickness, palisade tissue thickness, spongy tissue thickness, and the palisade-to-spongy tissue ratio of *F. mandshurica*, respectively. LMT, LMPT, LMPhT, and LMXyT represent main vein thickness, main vein parenchyma thickness, main vein phloem thickness, and main vein xylem thickness of *F. mandshurica*, respectively. Color intensity indicates the level of significance: darker colors represent stronger significance, whereas lighter colors indicate weaker correlations. * indicates significance at *p* < 0.05, and ** indicates significance at *p* < 0.01.

*P. koraiensis* mainly exhibited coordinated relationships between needle anatomical and photosynthetic traits, whereas *F. mandshurica* showed stronger integration among leaf morphology, anatomy, and photosynthetic performance (Figure 5). In *P. koraiensis*, most needle anatomical variables were significantly positively correlated with one another and Pn, Tr, and Gs, but negatively correlated with Ci (Figure 5A). In contrast, mesophyll ratio and Ci showed the opposite pattern, being negatively correlated with Pn, Tr, and Gs and other needle anatomical traits. LW and LA were strongly positively correlated with Tr, Gs, and most needle tissue areas, whereas SLA was negatively correlated with Pn, Tr, Gs, resin duct lumen area, xylem area, and xylem ratio.

In *F. mandshurica*, leaf morphology, photosynthetic traits, and anatomical characteristics were even more tightly coordinated (Figure 5B). The thicknesses of all main vein tissues were highly significantly and positively correlated with one another. Leaf thickness, palisade tissue thickness, and all main vein structural traits were positively correlated with Pn, Tr, and Gs. In addition, leaf morphological traits and photosynthetic parameters were all significantly positively correlated. Similar to *P. koraiensis*, Ci showed the opposite pattern, being negatively correlated with leaf size, other photosynthetic traits, WUE, and several anatomical characteristics.

**Figure 6:**
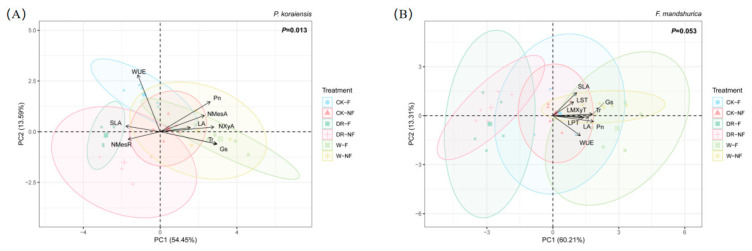
PCA of leaf traits in *Pinus koraiensis* (**A**) and *Fraxinus mandshurica* (**B**) in response to different water–fertilization conditions.

Furthermore, principal component analysis (PCA) showed that the first two axes explained most of the variation in leaf morphological, physiological, and anatomical traits in both species, but their overall response patterns differed (Figure 6). In *P. koraiensis*, PC1 and PC2 explained 54.45% and 13.59% of the variation, respectively (68.04% in total), and treatment separation was significant (*p* = 0.013). PC1 was positively associated with Pn, Tr, Gs, LA, mesophyll area, and xylem area, but negatively associated with SLA and mesophyll ratio. Water-addition treatments were distributed mainly on the positive side of PC1, whereas drought treatments were located on the negative side, indicating that high water availability promoted leaf expansion, gas exchange, and conductive tissue development, while drought favored a more conservative leaf strategy.

In *F. mandshurica*, PC1 and PC2 explained 60.21% and 13.31% of the variation, respectively (73.52% in total), and treatment separation was close to significant (*p* = 0.053). PC1 mainly represented coordinated changes in leaf morphology, photosynthetic performance, and anatomical traits. LA, SLA, Pn, Gs, WUE, and several anatomical variables loaded in the same direction. Water-addition treatments were generally distributed on the positive side of PC1, while drought treatments were located on the negative side, indicating that greater water supply promoted leaf expansion, photosynthetic activity, and coordinated tissue development. Overall, treatment differentiation was more pronounced in *P. koraiensis*, suggesting greater sensitivity to coupled water–fertilizer effects, whereas trait coordination was stronger in *F. mandshurica*, indicating a more integrated response.

## 3. Discussion

### 3.1. Morphological Traits of Leaf Response to Water–Fertilization Conditions

Leaf morphology is one of the most sensitive and plastic traits through which plants respond to changes in water and nutrient availability [9,25]. In the present study, water availability was the primary driver of leaf morphological variation in both *P. koraiensis* and *F. mandshurica*. LA and LW increased significantly with increasing water supply in both species, indicating that adequate water availability promoted leaf expansion and thus enhanced light interception capacity. This pattern agrees with the evidence showing that drought generally constrains leaf expansion, whereas favorable water conditions promote the development of larger assimilatory surfaces [26,27]. However, the two species exhibited contrasting responses in SLA. In *P. koraiensis*, SLA increased under drought and decreased under high water supply, whereas the opposite trend was observed in *F. mandshurica*. Such divergence suggests fundamentally different resource-use strategies between the two species. The lower SLA of *F. mandshurica* under drought is consistent with a conservative strategy characterized by smaller or denser leaves that reduce transpirational water loss, whereas the increase in SLA under favorable water conditions reflects a shift toward greater light capture and rapid growth. By contrast, the increase in SLA in *P. koraiensis* under drought may indicate a species-specific adjustment that maintains minimum photosynthetic surface area under stress, while the decline in SLA under high water supply suggests a tendency to construct thicker and denser needles once water limitation is removed. Similar species-specific or provenance-specific SLA responses to drought have been reported in both herbaceous and woody plants [27,28], highlighting that leaf morphological adjustment does not follow a single universal pattern but depends on functional type and evolutionary strategy.

Fertilization further modified leaf morphological responses, but its effects differed between the two species. In *P. koraiensis*, fertilization significantly affected LA and showed a clear interaction with water regime, particularly under drought, where N and P addition alleviated the inhibition of leaf expansion caused by water limitation. This suggests that improved nutrient availability can partially offset drought-induced constraints on leaf growth by supporting cell division, tissue expansion, and carbon–nutrient coordination [29,30]. In contrast, leaf morphology in *F. mandshurica* was largely insensitive to fertilization, indicating that this species remained more strongly water-driven than nutrient-driven at the morphological level. These interspecific differences imply that *P. koraiensis* is more responsive to water–fertilizer coupling, whereas *F. mandshurica* seedlings rely more directly on water availability to regulate leaf structural development. This interpretation is consistent with previous studies showing that nutrient supply can promote leaf expansion and biomass allocation, but that such effects are often contingent on water status and differ among species with contrasting ecological strategies.

### 3.2. Photosynthetic Parameters of Leaf Response to Water–Fertilization Conditions

The gas-exchange responses of the two species also reflected the dominant role of water availability. In both *P. koraiensis* and *F. mandshurica*, Pn, Tr, and Gs increased significantly under high-water conditions, whereas Ci declined. This pattern is consistent with the view that a sufficient water supply maintains stomatal opening, facilitates CO_2_ diffusion into the mesophyll, and thereby supports higher photosynthetic carbon assimilation [31,32]. Under drought, however, both species showed decreases in Pn and Gs accompanied by an increase in Ci, suggesting that drought reduced photosynthesis not only through stomatal closure but also through non-stomatal limitations under severe stress [16]. Such responses have been widely documented and are often interpreted as evidence that mesophyll or chloroplast function becomes impaired once drought exceeds a threshold [33]. It should be noted that plant water status and PSII activity were not directly quantified in this study. Although gas-exchange and anatomical traits provided indirect evidence of seedling responses to drought, direct measurements such as relative water content, leaf water potential, osmotic potential and chlorophyll fluorescence parameters would be needed to more fully elucidate the physiological mechanisms underlying drought adaptation under different water and nutrient conditions. WUE also differed markedly between the two species. *P. koraiensis* displayed a hump-shaped response, with maximum WUE at intermediate soil moisture, indicating the presence of a clear optimum in water use. Under high water supply, the increase in Tr outpaced that of Pn, reducing WUE and implying excessive water loss. It may be due to reduced soil aeration and potential root-zone hypoxia for *P. koraiensis* seedlings under the highest soil moisture treatment. By contrast, WUE in *F. mandshurica* continued to increase with water availability, suggesting that this fast-growing broad-leaved species was better able to convert additional water supply into carbon gain under moist conditions.

Nitrogen and phosphorus fertilization mainly affected photosynthesis by improving nutritional status and modifying water-use characteristics, but their effects were strongly dependent on the water regime [34]. In *P. koraiensis*, fertilization had a highly significant effect on WUE and markedly improved both WUE and Pn under drought, indicating that nutrient supply alleviated the physiological constraints imposed by water deficit. This supports the idea that N and P addition can sustain photosynthetic enzyme synthesis, ATP production, and metabolic activity under stress, thereby partly buffering drought-induced declines in gas exchange [35,36]. In *F. mandshurica*, fertilization significantly affected Pn, Ci, and WUE, but the interaction between water and fertilization was significant only for Pn, suggesting that nutrient addition improved photosynthetic performance mainly under well-watered conditions by optimizing stomatal behavior and CO_2_ use efficiency. These results indicate that the effects of fertilization on photosynthesis are not independent of water supply. Instead, nutrient addition tends to enhance physiological performance when water conditions allow the additional nutrients to be effectively utilized, while under stronger water limitation, its effect is either reduced or expressed mainly through improvements in efficiency rather than absolute gas exchange.

### 3.3. Anatomical Responses of Leaf to Water–Fertilization Conditions

Both *P. koraiensis* and *F. mandshurica* showed strongly water-driven leaf anatomical responses, whereas fertilization had only minor effects. Under higher water supply, *P. koraiensis* mainly enhanced conductive tissues: needle xylem area and xylem ratio increased relative to drought, indicating greater investment in transport capacity. In contrast, *F. mandshurica* showed coordinated expansion of both assimilatory and vascular tissues. Under drought, the two species adopted different strategies. *P. koraiensis* maintained a higher relative mesophyll proportion despite reduced needle size, suggesting a conservative strategy to preserve basic photosynthetic function. By contrast, *F. mandshurica* showed decreases in leaf thickness and palisade development under drought, indicating that the imposed water deficit exceeded its structural adjustment capacity and directly suppressed leaf development. Fertilization did not alter these water-dominated patterns: in *P. koraiensis*, it only slightly adjusted mesophyll and vascular proportions, whereas in *F. mandshurica,* it mainly affected main vein parenchyma thickness and often in a direction opposite to that of water supply.

## 4. Materials and Methods

### 4.1. Study Site and Plant Material

The experiment was conducted in a greenhouse at the Mao’ershan Experimental Forest Farm of Northeast Forestry University (127°29′–127°44′ E, 45°14′–45°29′ N). This region is characterized by a temperate continental monsoon climate. The annual rainfall in this region amounts to approximately 700–750 mm, and the average annual temperature ranges from 2.7 to 2.9 °C.

The study material consisted of one-year-old container seedlings of *P. koraiensis* and *F. mandshurica*. Seeds originating from Lushuihe Korean Pine Mother Forest (127°29′–128°02′ E, 42°20′–42°49′ N) were stratified at 5 °C for five months and sown in the greenhouse in April 2023 for cultivation. In mid-April 2024, one-year-old seedlings were transplanted into the pots (15 × 10 × 18 cm, upper diameter × bottom diameter × depth) with one individual per pot. Each pot was filled with approximately 700 g of substrate consisting of peat and perlite mixed at a 4:1 (*v*/*v*) ratio. The bulk density of the substrate is 0.304 g/cm^3^, the pH value is 6.0, the total nitrogen content is 15.90 ± 0.33 mg/g, the total phosphorus content is 2.28 ± 0.02 mg/g, and the total potassium content is 6.40 ± 0.10 mg/g. After transplantation, seedlings were allowed to recover for 15 days before treatments were performed.

### 4.2. Experimental Design

The experiment followed a completely randomized, two-factor factorial design with water availability and fertilization as fixed factors. Water treatments comprised three levels based on soil volumetric water content (VWC): drought (DR), VWC maintained at ~13% (ca. 30% of field capacity); Control (CK), VWC maintained at ~26% (ca. 55% of field capacity); and water addition (W), VWC maintained at ~39% (ca. 85% of field capacity). Soil moisture was regulated using a combination of pot weighing and a soil multiple parameter recorder (SN-3002-TRREC-N01, Shandong VEMSEE Technology Co., Ltd., Jinan, China). The target pot weights were determined in advance based on the dry weight of the potting substrate and the amount of water required to reach each treatment level. Repeated measurements indicated that the instrument readings were approximately equivalent to VWC. Then, the soil volumetric water content (VWC) was converted to field capacity using a formula. Based on the corresponding relationship between field capacity and VWC, soil VWC was subsequently monitored regularly every 3 days with the instrument. The water content of treatments below the target level was replenished by adding water to return each pot to its target weight.

Within each watering regime, pots were assigned to two fertilization treatments: no fertilization (NF: 0 g N, 0 g P per plant) and N-P fertilization (F: 1 g N and 0.5 g P per plant in total). The total fertilizer amount was divided equally among the eight applications, from 2 May to 15 August 2024, at two-week intervals. (NH_4_)_2_SO_4_ was used as the N source and KH_2_PO_4_ as the P source. K_2_SO_4_ was added as needed to equalize the K input among treatments. For each tree species, a total of 360 pots of seedlings were assigned to six water–fertilization treatment combinations, with 60 replicate pots for each treatment. Weeds emerging in the pots were removed manually throughout the experiment.

### 4.3. Measurement of Leaf Morphological Traits

Leaf area, leaf length, and leaf width were measured using a scanner (CanoScan LiDE 120, Canon Inc., Tokyo, Japan) in combination with ImageJ 1.36b software (National Institutes of Health, Bethesda, MD, USA). The leaves were then oven-dried at 65 °C to constant weight (precision = 0.0001 g), and specific leaf area was calculated: specific leaf area (cm^2^·g^−1^) = leaf area/leaf dry mass.

### 4.4. Photosynthetic Parameters Measurement

Gas exchange parameters were measured on mornings between 09:00 and 12:00. For each treatment, five seedlings of each tree species were randomly selected. On each *P. koraiensis* seedling, five healthy current-year needles were measured, whereas on each *F. mandshurica* seedling, one fully expanded leaf was selected for measurement using a photosynthesis system LI-6800 (LI-COR Biosciences, Lincoln, NE, USA). The following parameters were recorded: net photosynthetic rate (Pn, μmol·m^−2^·s^−1^), transpiration rate (Tr, mmol·m^−2^·s^−1^), stomatal conductance (Gs, mmol·m^−2^·s^−1^), and intercellular CO_2_ concentration (Ci, μmol·mol^−1^). Needle intrinsic water-use efficiency (iWUE, μmol·mmol^−1^) was calculated as the following equation: iWUE = Pn/Tr.

### 4.5. Anatomical Analysis of Leaf

Current-year needles of *P. koraiensis* and *F. mandshurica* were cut into ~5 mm segments and fixed in FAA solution, then stored at 4 °C. Paraffin sections were prepared following standard histological procedures and after Safranin–Fast Green staining, samples were mounted with neutral resin to obtain permanent slides. For each slide, five intact cross-sections of a needle were selected. Sections were photographed using a microscope imaging system (Axio Observer 3, Zeiss, Oberkochen, Germany), and images were saved for subsequent analysis. For each treatment, five slides were selected for recordation. ImageJ software was used to quantify anatomical traits, including cross-section area (NCroA), resin duct area (NResA), mesophyll area (NMesA), phloem area (NPhA), and xylem area (NXyA) in needle; leaf thickness (LT), palisade tissue thickness (LPT), spongy tissue thickness (LST), palisade-to-spongy tissue ratio (P/S), main vein thickness (LMT), main vein parenchyma thickness (LMPT), main vein phloem thickness (LMPhT), and main vein xylem thickness (LMXyT) in leaf of *F. mandshurica*. In addition, the ratios of anatomical structure were calculated to characterize the proportion changes under the water regime and N-P fertilization.

### 4.6. Data Analysis

Data were organized using Microsoft Excel 2020. Two-way analysis of variance (ANOVA) was performed using SPSS 27.0 for Windows (IBM Corp., Armonk, NY, USA) to test the effects of water regime and N-P fertilization, as well as their interaction, on all measured traits of *P. koraiensis* and *F. mandshurica* seedlings. LSD’s multiple test was employed for multiple comparisons to determine significance. Pearson’s correlation coefficients were calculated among gas-exchange traits (Pn, Tr, Gs, Ci, WUE), foliar morphological and leaf anatomical traits. In addition, principal component analysis (PCA) was performed on the combined set of physiological, morphological and anatomical variables to summarize the main axes of trait covariation and to visualize the separation of treatments in multivariate trait space. PCA and correlation analyses were conducted in R (version 4.2.2) using the base functions and the package corrplot for visualization. Figures were prepared using Origin 2021 (OriginLab Corporation, Northampton, MA, USA) and GraphPad Prism 8.0.2 (GraphPad Software, San Diego, CA, USA).

## 5. Conclusions

Water availability was the dominant driver of leaf morphology, photosynthetic performance, and leaf anatomy in both species, whereas fertilization mainly modified these responses in a water-dependent manner. *P. koraiensis* showed a non-linear response to increasing water supply, with water-use efficiency peaking at intermediate soil moisture and conductive tissues responding more strongly to coupled water–nutrient variation. In contrast, *F. mandshurica* showed more coordinated increases in leaf expansion, photosynthetic activity, and assimilatory–vascular development under improved water conditions. These contrasting response patterns suggest that the two species may differ substantially in regeneration performance and ecological functioning under future fluctuations in water availability and increasing nitrogen and phosphorus deposition.

## Figures and Tables

**Table 1:** Leaf morphological traits of *Pinus koraiensis* seedlings under different water and nitrogen–phosphorus fertilization treatments.

Fer	Water	LA (cm^2^)	SLA (cm^2^·g^−1^)	LW (mm)
NF	DR	5.48 ± 0.81 Ba	79.59 ± 8.46 Aa	0.87 ± 0.11 Ba
	CK	8.26 ± 2.22 Aa	74.36 ± 6.51 Aa	1.08 ± 0.18 Aa
	W	7.01 ± 1.55 ABb	74.55 ± 4.75 Aa	1.07 ± 0.1 Aa
F	DR	7.54 ± 1.42 Ba	89.89 ± 14.77 Aa	0.88 ± 0.09 Ca
	CK	7.17 ± 0.96 Ba	80.46 ± 7.09 ABa	1.03 ± 0.07 Ba
	W	10.08 ± 2.55 Aa	74.69 ± 5.18 Ba	1.19 ± 0.03 Aa
*p*-values	WT	*	*	**
	Fer	*	ns	ns
	WT × Fer	*	ns	ns

Note: The table shows leaf area (LA), specific leaf area (SLA), and leaf width (LW) of *Pinus koraiensis* under different water regimes and nitrogen–phosphorus fertilization treatments. WT indicates water treatment, Fer indicates fertilization treatment, and WT × Fer indicates the interaction between water treatment and fertilization treatment. Data are presented as mean ± SE (*n* = 5). Different uppercase letters after the values indicate significant differences among water treatments under the same fertilization condition (*p* < 0.05), whereas different lowercase letters indicate significant differences between fertilization treatments under the same water condition (*p* < 0.05). * indicates *p* < 0.05; ** indicates *p* < 0.01; ns indicates not significant.

**Table 2:** Leaf morphological traits of *Fraxinus mandshurica* seedlings under different water and nitrogen–phosphorus fertilization treatments.

Fer	Water	LA (cm^2^)	SLA (cm^2^·g^−1^)	LW (mm)
NF	DR	20.82 ± 2.61 Ba	231.33 ± 15.28 Aa	4.02 ± 0.32 Ba
	CK	26.51 ± 2.61 Aa	242.36 ± 14.66 Aa	4.56 ± 0.22 Aa
	W	28.28 ± 3.17 Aa	252.48 ± 11.77 Aa	4.6 ± 0.28 Aa
F	DR	22.33 ± 1.48 Ba	224.33 ± 23.74 Aa	4.25 ± 0.23 Ba
	CK	25.42 ± 1.34 Ba	245.71 ± 14.75 Aa	4.42 ± 0.11 Ba
	W	30.35 ± 3.36 Aa	245.16 ± 15.35 Aa	4.93 ± 0.44 Aa
*p*-values	WT	**	*	**
	Fer	ns	ns	ns
	WT × Fer	ns	ns	ns

Note: Different uppercase letters after the values indicate significant differences among water treatments under the same fertilization condition (*p* < 0.05), whereas different lowercase letters indicate significant differences between fertilization treatments under the same water condition (*p* < 0.05). * indicates *p* < 0.05; ** indicates *p* < 0.01; ns indicates not significant. Data are presented as mean ± SE (*n* = 5).

## Data Availability

All data generated or analyzed in this study are included in this article and its Supplementary Information.

## Supplementary Materials

The following supporting information can be downloaded at: https://www.mdpi.com/article/10.3390/plants15132053/s1, Figure S1: Leaf anatomical structure of *P*. *koraiensis* seedlings under water regime and nitrogen-phosphorus addition; Figure S2: Anatomical structure on both sides of leaf midvein of *F. mandshurica* seedlings under water regime and nitrogen-phosphorus addition; Figure S3: Leaf main veins anatomical structure of *F. mandshurica* seedlings under water regime and nitrogen-phosphorus addition.
