# Supplementary material for: Contrasting Leaf Adaptation Strategies of Pinus koraiensis and Fraxinus mandshurica Under Water and Nutrient Variation
Source: Plants (Basel). 2026 Jul 2;15(13):2053. doi: 10.3390/plants15132053 (PMC13363737; doi:10.3390/plants15132053)
Supplement: Supplementary file 1 [file plants-15-02053-s001.zip › plants-4361383-supplementary.pdf]

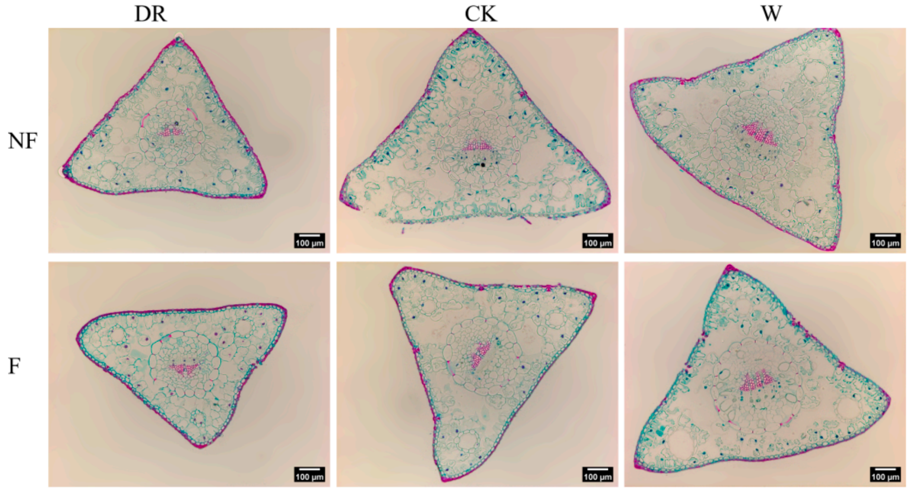

Figure S1. Leaf anatomical structure of *P. koraiensis* seedlings under water regime and nitrogen-phosphorus addition.

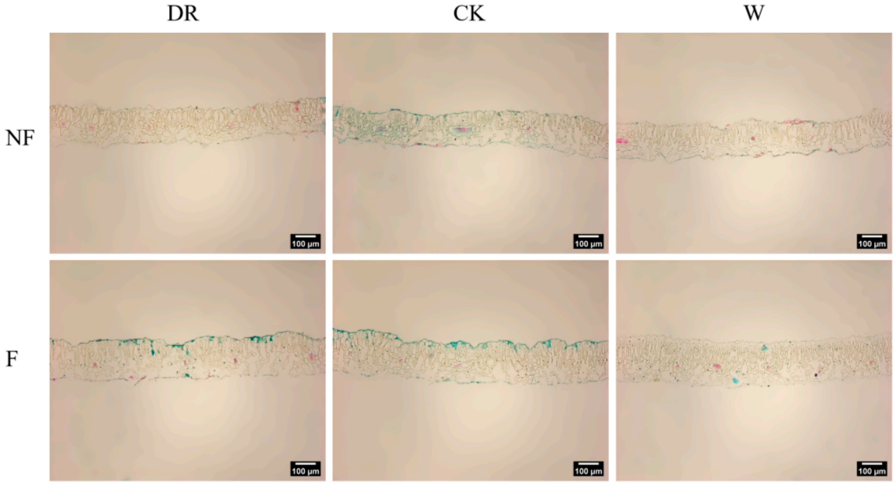

Figure S2. Anatomical structure on both sides of leaf midvein of *F. mandshurica* seedlings under water regime and nitrogen-phosphorus addition.

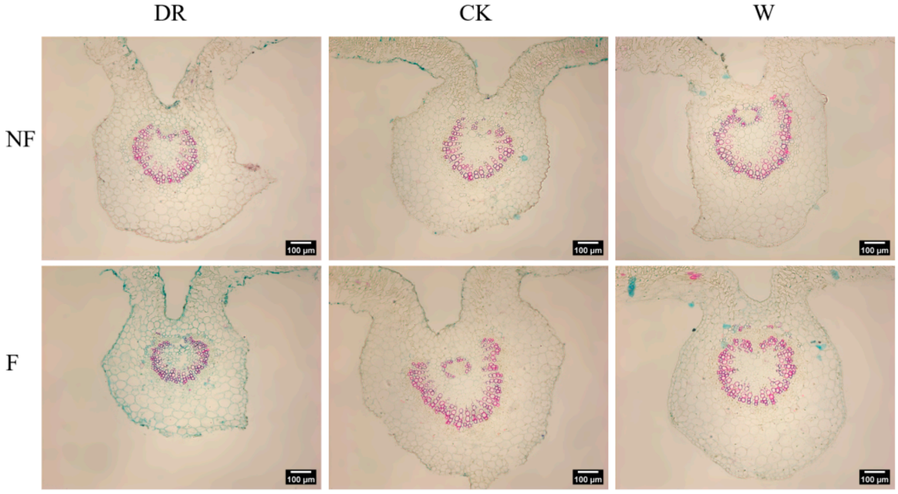

Figure S3. Leaf main veins anatomical structure of *F. mandshurica* seedlings under water regime and nitrogen-phosphorus addition.
